# Extending donor criteria: successful lung transplantation post *ex vivo* back table donor pulmonary artery thrombolysis

**DOI:** 10.1093/jscr/rjad367

**Published:** 2023-06-22

**Authors:** Samuel Jacob, Pankaj Garg, Magdy El-Sayed Ahmed, Ian A Makey

**Affiliations:** Department of Cardiothoracic Surgery, Heart and Lung Transplantation program, Mayo Clinic, Jacksonville, FL, USA; Department of Cardiothoracic Surgery, Heart and Lung Transplantation program, Mayo Clinic, Jacksonville, FL, USA; Department of Cardiothoracic Surgery, Heart and Lung Transplantation program, Mayo Clinic, Jacksonville, FL, USA; Department of Cardiothoracic Surgery, Heart and Lung Transplantation program, Mayo Clinic, Jacksonville, FL, USA

## Abstract

Pulmonary embolization in donor lungs is a common finding and found in up to 38% of cases. To expand the pool of organs, transplant centers now utilize lungs, from increased risk donors, that may have pulmonary embolic disease. Modalities of clearing pulmonary artery embolisms are critical to reduce the prevalence of primary graft dysfunction post transplantation. There have been anecdotal cases of pulmonary embolectomy pre and post organ procurement or *in vivo* and *ex vivo* thrombolytic therapy performed in donors with massive pulmonary emboli. We report for the first time therapeutic *ex vivo* thrombolysis on the back table without Ex Vivo Lung Perfusion (EVLP), followed by successful transplantation.

## INTRODUCTION

Accepting donors with pulmonary embolisms (PEs) is becoming a common practice in busy centers to extend donor lungs availability, which include using lungs from older donors, living donors, reversible underlying pathology and donor after circulatory death. Most of these cases reported successful outcome [1, 2].

PE is a frequent finding in organ donors [3], as donor lungs are prone to PE from acute deep vein thrombosis (DVT), dislodging of thrombus from central venous line and *in situ* thrombus formation due to hypothermia during hospitalization or during organ harvesting [4, 5]. Recovery of macroscopic thrombi during retrograde preservation flush is associated with worse outcomes after lung transplantation [8]. Although there have been cases of pulmonary embolectomy after organ procurement or thrombolytic therapy performed in donors with massive PE [4, 6, 7], we report, for the first time, therapeutic *ex vivo* thrombolysis on back table followed by clinical transplantation.

## CASE REPORT

**Figure 1:**
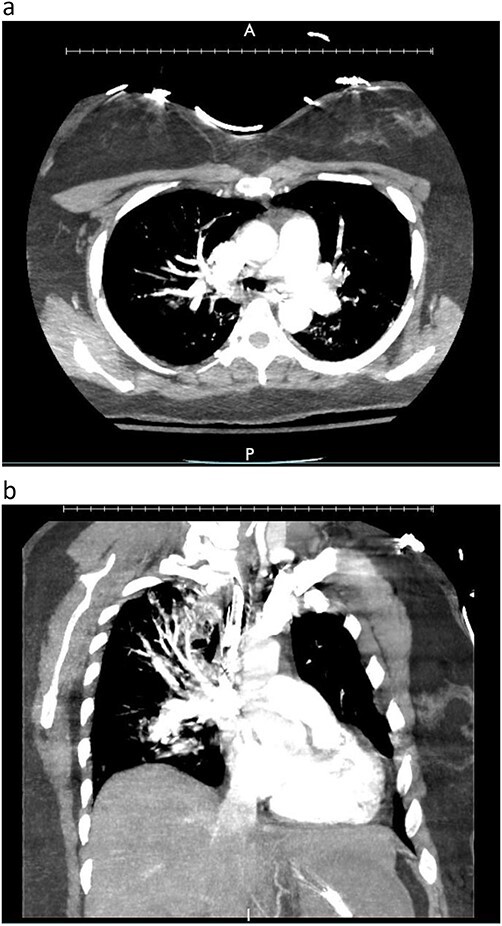
Donor angio CT scan: (**a)** filling in the left PA, (**b)** reduction of blood supply of the left lung.

A woman in her mid-30s had hypoxic brain injury after cardiac arrest from drug overdose and was declared brain dead 4 days after hospitalization. PaO_2_ was 466 mm Hg with a fraction of inspired oxygen of 1.0 and positive end-expiratory pressure of 5 cm H_2_O. Chest computed tomography (CT) angiography showed a subtle filling defect in the left pulmonary artery (LPA) (Fig. 1). Intraoperative bronchoscopic findings were unremarkable. On visual inspection, the lungs appeared normal and had adequate compliance. We accepted and procured the lungs en bloc. Retrograde flushing evacuated several small clots from the pulmonary arteries. Both lungs were packed and transported as usual.

**Figure 2:**
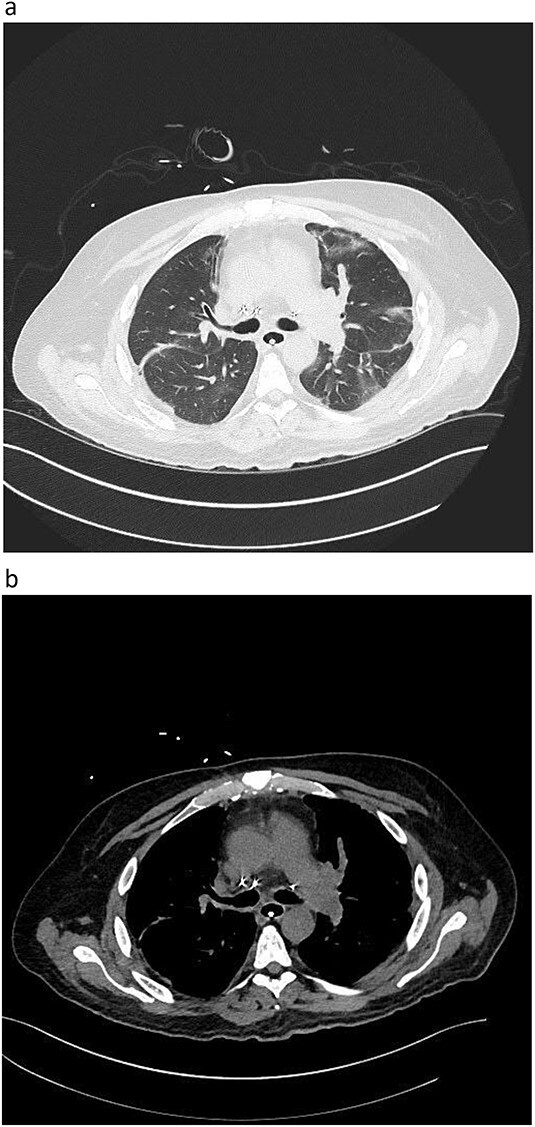
Postoperative graft CT scan: recipients had bilateral lung reduction due to over size.

**Figure 3:**
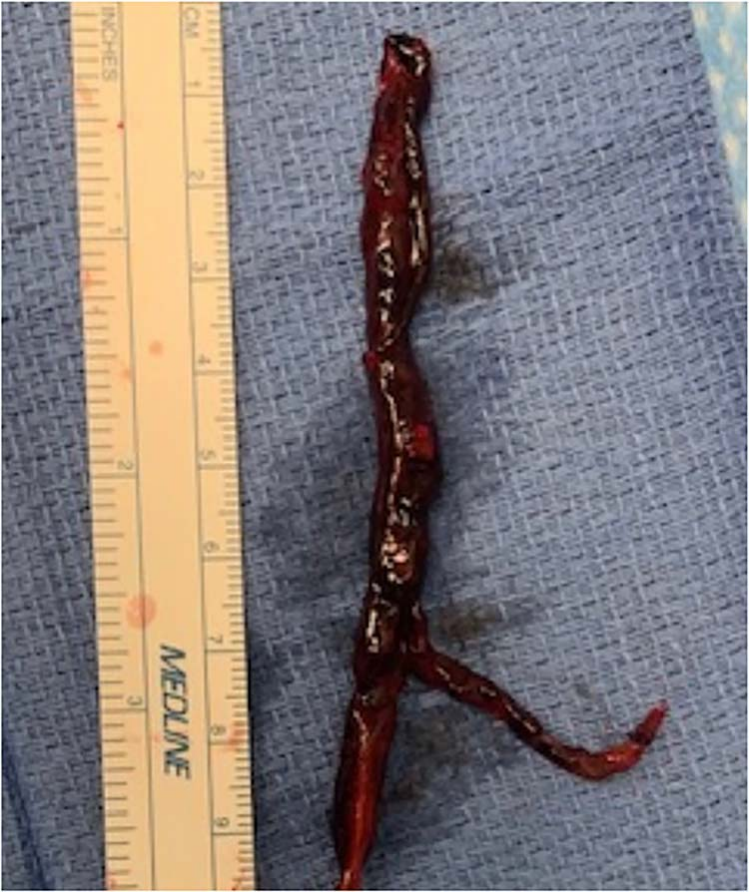
Extracted LPA thrombus.

During back-table preparation at the recipient hospital, a large partially adherent thrombus was observed in the LPA, suggesting a subacute process. We gently peeled and removed a 10-cm thrombus from the LPA wall (Fig. 3). Because we were unsure whether more thrombi were present in the left lung, we performed thrombolysis by injecting 4 mg of recombinant tPA (alteplase) into the LPA and let it dwell for 10 min before a second retrograde flush with 2 L of PERFADEX Plus (XVIVO) solution, which removed many more clots. Bilateral sequential lung transplant was performed in a 50-year-old female recipient who was on extracorporeal membrane oxygenation because of COVID-19–associated pneumonia. After transplant, she was weaned from extracorporeal membrane oxygenation and had an uneventful recovery. She was discharged 30 days after transplant (Fig. 2).

## DISCUSSION

It is very common for the donor lungs to contain shower of small, microscopic and macroscopic pulmonary emboli [9]. These emboli are believed to originate from indwelling central venous catheter or pulmonary artery catheter, or lower extremities DVT. Donors are also prone to develop *in situ* pulmonary artery thrombi also during their hospitalization as well as during and after the procurement of the lungs. These thrombi may develop in the pulmonary circulation due to sudden reduced activity of the pulmonary fibrinolytic system due to the exposure to cold solution and topical cooling [10].

It has been our observation to have significant blood return from the pulmonary arties on back table flush despite flushing the lungs with 4 L of cold Perfadex solution before procurement.

It is routine practice to back flush the pulmonary circulation with 2 L of Perfadex solution after the procurement and small thrombi in the donor pulmonary arteries are easily flushed with this technique [7]. It is however, not uncommon to retrieve long clots during the flush. In these patients, one is never sure of complete removal of the clots. Studies have also shown that recovery of macroscopic thrombi during retrograde preservation flush is associated with worse outcomes after lung transplantation [9]. Therefore, lung donation after massive PE in donor lungs is usually discouraged. However, there are few case reports of successful thrombolysis of the donor lungs prior to the donation or removal of the thrombus after the procurement of the lungs and good outcome after the implantation [9–11]. In a case report by Machuca *et al*. [6] from Toronto General Hospital, authors performed the *ex vivo* thrombolysis for the PE on EVLP circuit. Authors observed the gradual reduction in pulmonary artery pressure and significantly improved compliance after the thrombolysis. Lungs were successfully transplanted with good outcome. This report paved the way that it is feasible to do *ex vivo* thrombolysis.

Our technique of injecting the urokinase down the branch pulmonary arteries and wait period of 15 min followed by repeat backflush is sufficient in lysing and removing macroscopic clots. Further, that will add to the total ischemic time 20 min, which is not substantial and tolerable by preserved lungs. We recovered more clots on second retrograde flush after the thrombolysis. We are not sure whether this was the result of thrombolysis alone or combined effect of thrombolysis and mechanical mobilization of the thrombi due to flush.. Our report shows that lungs which have significant amount of macroscopic clot burden on retrograde flush should be thrombolyzed prior to implantation. Whether the isolated finding of macroscopic clots during the retrograde flush would benefit from back table thrombolysis in all instances, for example to lyse distal clots that cannot be surgically extracted, is a question that warrants further exploration. Nevertheless, our approach seems worthy of consideration.

One concern was the potential deleterious activity of tissue plasminogen activator after reperfusion of the lungs in the recipient. Tissue plasminogen activator is safe and a routinely used thrombolytic agent in patients with acute myocardial infarction and stroke [12]. Further, additional flush 10 min after instillation of tissue plasminogen activator is expected to clear the medication from the pulmonary vasculature. In our recipient there were no signs of fibrinolytic complication such as bleeding after the lung transplantation. Uneventful recovery of our patient without pulmonary complications or bleeding establishes the safety of the technique and exemplifies how the concept of personalized medicine for the donor organ can be applied in the field of lung transplantation.

## CONFLICT OF INTEREST STATEMENT

There is no conflict of interest.

## FUNDING

This research is not funded.

## DATA AVAILABILITY

Data is available upon request.
